# Multidimensional engineered metasurface for ultrafast terahertz switching at frequency-agile channels

**DOI:** 10.1515/nanoph-2021-0774

**Published:** 2022-02-22

**Authors:** Yuze Hu, Mingyu Tong, Siyang Hu, Weibao He, Xiang’ai Cheng, Tian Jiang

**Affiliations:** College of Advanced Interdisciplinary Studies, National University of Defense Technology, Changsha 410073, P. R. China; Beijing Institute for Advanced Study, National University of Defense Technology, Changsha 410073, P. R. China

**Keywords:** electromagnetically induced transparency, hybrid metasurface, terahertz metamaterials, ultrafast photoswitching

## Abstract

The ability to actively manipulate free-space optical signals by using tunable metasurfaces is extremely appealing for many device applications. However, integrating photoactive semiconductors into terahertz metamaterials still suffers from a limited functionality. The ultrafast switching in picosecond timescale can only be operated at a single frequency channel. In the hybrid metasurface proposed here, we experimentally demonstrate a dual-optically tunable metaphotonic device for ultrafast terahertz switching at frequency-agile channels. Picosecond ultrafast photoswitching with a 100% modulation depth is realized at a controllable operational frequency of either 0.55 THz or 0.86 THz. The broadband frequency agility and ultrafast amplitude modulation are independently controlled by continuous wave light and femtosecond laser pulse, respectively. The frequency-selective, temporally tunable, and multidimensionally-driven features can empower active metamaterials in advanced multiplexing of information, dual-channel wireless communication, and several other related fields.

## 1 Introduction

Ultrathin and tunable optical elements have considerable impact on various applications where the miniaturized size and constrained weight are unmatched by traditional optical components. Metasurfaces, the two-dimensional planar arrays of metamaterials, have shown outstanding and unusual properties by efficiently converting propagating light to local fields at an interface within thicknesses of several hundreds of nanometers [1], [2], [3], [4], [5]. The versatile manipulation of electromagnetic wave responses in terahertz frequency regimes has attracted considerable attention from global research communities because of its diverse applications, such as medical imaging [6, 7], nondestructive diagnostics [8], [9], [10], and next-generation wireless communication [11], [12], [13]. Indeed, most metasurfaces with strong light–material interaction offer a feasible solution to compensate for the lack of appropriate terahertz responses in naturally existing materials. Practical applications of functional-rich terahertz metasurfaces include tunable chirality emitter [14], polarization converters [15], multiplexing holograms [16], quarter and half-wave plates [17], planar lenses [18], invisibility cloaking [19], and biological sensing [20]. In the past decade, research efforts have been mainly devoted to the studies of actively-controlled terahertz waves. The active material integrated metasurfaces with a flexible tunability are termed as terahertz metadevices. This has attracted great interest in the development of a new terahertz device paradigm, such as spectrally tunable filters [21, 22], switchable polarizers [23], beam steerers [24], [25], [26], and high-performance information coding [27, 28]. The corresponding active materials are presented for the realization of terahertz metadevices, mainly including a plethora of semiconductors, superconductors, liquid crystals, deformable materials, and phase change materials (PCMs), with the external stimuli varying from optical, temperature, and electrical-to-mechanical approaches [29], [30], [31], [32], [33], [34]. However, most of these approaches are limited by a single functionality, such as one resonance mode switching, unchangeable modulation speed, and fixed operation frequency. The response of active materials to a single driving field is often a dilemma, which makes it increasingly difficult to achieve higher levels of terahertz control over multiple dimensions. Therefore, substantial efforts are still required to unleash the full versatility of metasurfaces.

Along with multipurpose functionalities, the switching speed is equally an important factor that affects the utilization of devices such as modulators or switches. Multiple studies have attempted to modulate metasurfaces with various semiconductors, including traditional semiconductors [31, 35], [36], [37], [38], [39], [40], superconductors [41], perovskites [42, 43], Weyl semimetals [44], topological insulators [45], and transition metal dichalcogenides [46]. Lim et al. designed and fabricated an amorphous Ge-based metasurface for all-optical terahertz switching on a picosecond timescale with the assistance of a defect-site-mediated ultrashort photocarrier lifetime [29]. Recently, a similar transmission amplitude switching behavior was further manifested in CVD-grown WSe_2_-functionalized metadevice [47]. However, a Fano-type resonance with multiple coupled metaatoms is required to compensate for the weak photoconductivity generated in defect-rich semiconductors. Hence, these methods impose a functional limitation on the narrow operating frequency that is, working frequency cannot be changed once the device is fabricated. By leveraging the concept of molecularization proposed by Jung [48], we have attempted to manipulate the electromagnetically induced transparency (EIT) resonance frequency via silicon bridges under optical pump injection [21]. Although multiple coupled metaatoms are molecularized simultaneously, only a single functionality of frequency agility is realized, and the switching time is longer than a nanosecond. On the other contrary, the temperature-triggered insulator-to-metal transition in PCMs has enormous potential to redesign active terahertz metamaterials. Currently, intensive research efforts have been devoted to PCM-hybrid terahertz metasurfaces for multifunctionality, including GST-based multilevel resonance switching [49, 50], VO_2_-hybrid amplitude/frequency/phase tuning [51], [52], [53], [54], [55], etc. Compared with the electrical-induced approach, the highly contrasting conductivity jump caused by external light illumination provides higher design flexibility, because no additional electrodes are required to connect each embedded island. This unique characteristic makes it possible to search for multidimensional controlled terahertz metadevices to fulfil the need for ultrafast all-optical terahertz switching at multi-frequency channels.

In this study, we experimentally demonstrate a strategy to create an ultrafast (picosecond) terahertz switching at frequency-agile channels based on the contrasting optical properties in the embedded active materials, operating as a novel metadevice with multidimensionally-controlled multifunctionalities. The key to achieving a frequency agility is using the light-induced phase transition in VO_2_-bridges to molecularize metaatoms that vastly shift the resonance frequency. The pattern of hybrid structures is exceptionally designed so that a significant Fano-type resonant feature is maintained in both the atomized and molecularized states. The local-field response of the Fano-type structure has the following feature that ensures ultrafast photoswitching performance: the capacitive gaps can be easily short-circuited by photocarriers, which leads to the obvious disappearance of the transparency window. A thin layer of amorphous Ge is then deposited onto the entire surface and simultaneously positioned in the capacitive gaps, which meets the basic requirement for ultrafast terahertz modulation by providing sufficient photocarriers with a sub-picosecond lifetime when subjected to optical pumps. By utilizing continuous waves (CWs) to accomplish molecularization and optical pumping to suppress resonances, we achieve ultrafast temporal terahertz switching at a specific frequency across a broadband tuning range.

## 2 Results and discussion

### 2.1 Realization of ultrafast terahertz switching at frequency-agile channels

The functionality of ultrafast and efficient terahertz switching can be readily achieved by actively tuning Fano-type resonance with semiconductor-hybrid metasurfaces, but the operating frequency cannot be adjusted once the metasurfaces are fabricated. Alternatively, by introducing the concept of molecularization and routing the connectivity between metaatoms, the resonance frequency of reconfigurable metasurfaces can be controlled. To integrate both functionalities into a single active metasurface, several stringent challenges need to be considered during the design of hybrid structures: (i) both resonance modes at two different frequencies provided by metaatoms must be sensitive to the generation of free carriers in the photoactive layer excited by an optical pump, (ii) one more degree of freedom is required to manipulate the resonance frequency shift by molecularizing metaatoms, and (iii) the properties of the two active materials must respond to their corresponding approaches of control independently, namely, one approach for ultrafast switching and the other for frequency shift, with no cross-talk between them.

**Figure 1:**
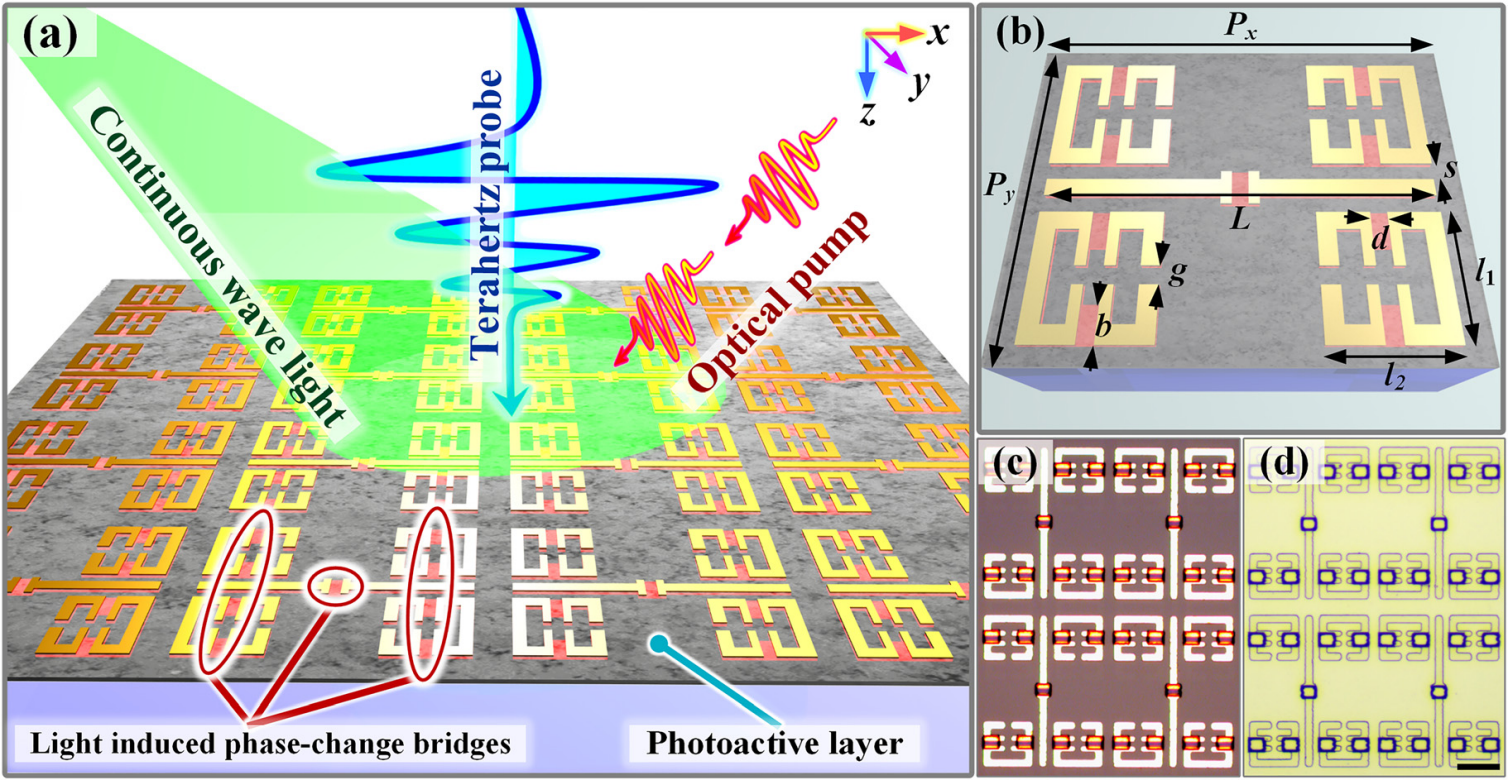
Working principle and characterization of dual-optically controllable metaphotonic devices. (a) A graphical illustration of the concept of the proposed functional metaatoms with ultrafast THz switching at selectable frequencies consisting of resonating metallic antennas hybridized with phase-change bridges and a photoactive layer. The bridges enable the light-induced molecularization of metaatoms to transit the operation frequency, whereas the photoactive layer provides pump-excited free carriers to short-circuit metaatoms with ultrafast switching speed. The incident THz wave polarized along the *x* direction is inserted into the metasurface propagating along the *k*_*z*_ direction. (b) A 3D artistic illustration of metaatoms within a unit cell, which consists of multiple coupled resonators with geometrical parameters: *L* = 100 µm, *l*_1_ = 35 µm, *l*_2_ = 30 µm, *s* = 5 µm, *b* = 10 µm, *d* = 4 µm, *g* = 5 µm, *P*_*x*_ = 120 µm, and *P*_*y*_ = 95 µm. Here, the thicknesses of the gold metamaterial, VO_2_-bridges, Ge photoactive layer, and sapphire substrate are 200 nm, 100 nm, 200 nm, and 500 µm, respectively. Reflective optical microscope images of the hybrid meta-atoms (c) before and (d) after the deposition of a 200-nm-thick Ge film. Scale bar: 30 µm.

Through the beneficial use of giant photoconductive sensitivity in EIT analogy metaatom systems, we first propose an EIT metasurface with broad band resonance frequency tunability, as shown in Figure 1(a). The effective length of cut-wire resonators (CWRs) and split ring resonators (SRRs) can be greatly extended by light induced phase-change bridges. In principle, unlike the simple dipole mode, the EIT analogy in optical metamaterials is generated by the destructive interactions between two pathways of |0⟩ → |1⟩ and |0⟩ → |1⟩ → |2⟩ → |1⟩ (ground state is |0⟩) to mimic a coherent three-level quantum system [56–58]. Here, two SRRs aside a CWR constitute a complete EIT platform, wherein the terahertz incidence directly drives the dipole moment in the CWR with a Lorentz line shape and then indirectly excites the dark LC mode in the SRRs by near-field coupling (referred to as EIT-I system). Thus, a transparency window occurs at the frequency determined by a short CWR and small SRRs. Metaatom molecularization requires multiple phase-transition bridges embedded into metaatoms to simultaneously interconnect two EIT-I systems; thus, leading to the formation of an EIT-II system consisting of one longer CWR and four larger SRRs. With the disappearance of the transparency window of the EIT-I, the EIT-II system can induce a new transparency window at another frequency. Such feature significantly promotes the realization of frequency agility. With the aid of a photoactive layer covering the entire surface, the SRR gaps in both the original and shifted EIT systems can be short-circuited effectively once photocarriers are excited. Regarding the adoption of active materials, the phase-change bridges are made of VO_2_, and the photoactive layer is made of amorphous Ge. VO_2_-bridges can transit from an insulator state to a metallic state via CW light illumination. Amorphous Ge has the potential for use in ultrafast all-optical switching owing to the ultrashort lifetime of its photocarriers when injected by femtosecond pulses. The possibility for the two optical approaches to manipulate the corresponding functionalities (frequency-agility and ultrafast amplitude switching) independently without cross-talk stems from the broad dependency of temperature-sensitive VO_2_ phase transition on the average optical power in CW light, and the photocarrier excitation in nanocrystalline Ge is determined by the peak optical power of the pulses.

By using the aforementioned geometrical and physical structures, the detailed geometric sizes within one-unit cell are optimized as shown in Figure 1(b). The metaatoms are made of gold and are equally spaced in the *x*- and *y*-directions with periodicities *P*_*x*_ = 120 µm and *P*_*y*_ = 95 µm, respectively. A more detailed and elementary description in Figure 1(b) is provided as *L* = 100 µm, *l*_1_ = 35 µm, *l*_2_ = 30 µm, *s* = 5 µm, *b* = 10 µm, *d* = 4 µm, and *g* = 5 µm. The central CWRs and side SRRs of the proposed metaatoms are bridged by nine VO_2_ islands (red patches). A thin layer of amorphous Ge film (gray layer) was deposited on top of a 500-µm-thick sapphire substrate. To prevent the possibility of THz wave leakage, the device occupies an area of 6 × 6 mm^2^ consisting of 3150 unit cells, which is much larger than the THz spot with a diameter of 2 mm. The sample was fabricated using a standard UV lithography technique and the various components were revealed with a microscope, as shown in Figure 1(c) and (d) before and after the deposition of the Ge film, respectively. All the VO_2_-bridges were successfully connected to the corresponding resonators, and the Ge film uniformly covered the entire surface to ensure filling in the gaps of the SRRs.

**Figure 2:**
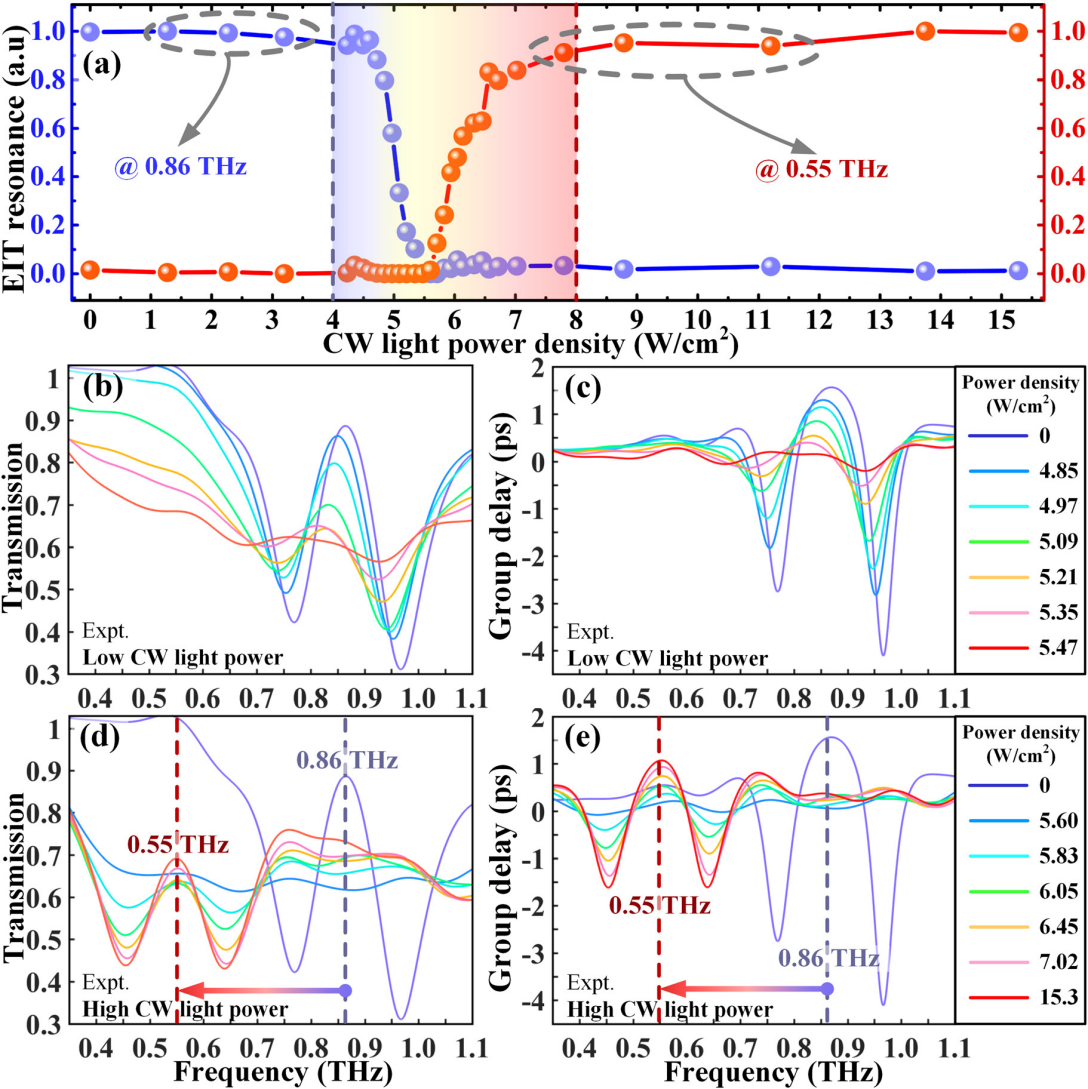
Experimental demonstration of THz spectrum responses as the CW light induced molecularization occurs. (a) Measured transmission amplitude of the normalized EIT resonance strength with the applied CW light density ranging from 0 to 15.3 W/cm^2^ for the EIT peak centered at 0.86 THz (blue curve) and 0.55 THz (red curve), respectively. Experimental (b) transmission and (c) group delay spectra of the metadevice with a low CW light power ranging from 0 to 5.47 W/cm^2^, leading to the disappearance of the EIT resonance at 0.86 THz. (d) and (e) In contrast, the corresponding THz dispersion spectra in the high CW light power case from 5.6 to 15.3 W/cm^2^ demonstrate the formation of a new EIT resonance at 0.55 THz, resulting in the molecularization of metaatoms and broadband resonance frequency shift.

**Figure 3:**
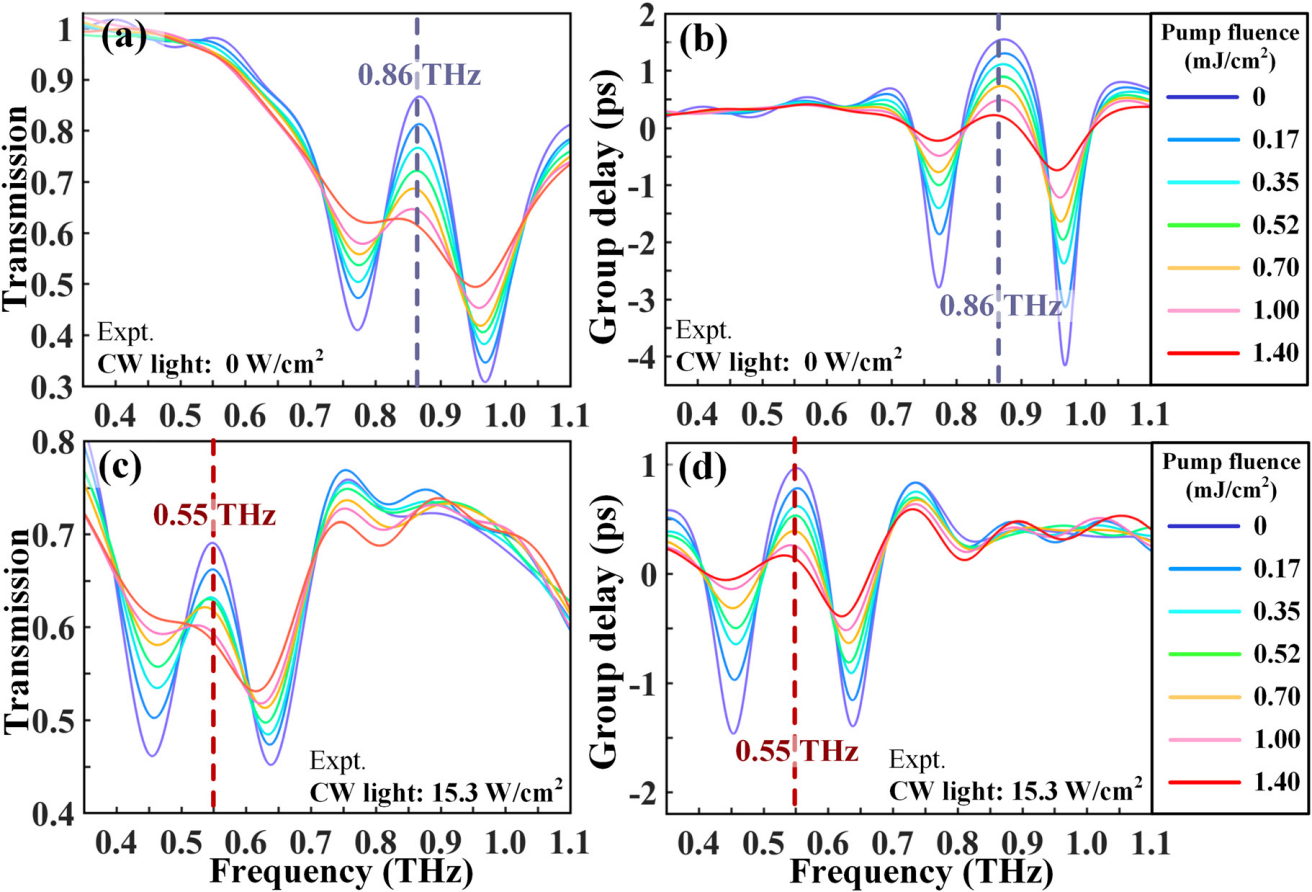
Controlling THz transmission/group delay spectrum via the femtosecond pulses impinging on the hybrid metadevice before and after the metaatom molecularization. Experimental (a) transmission and (b) group delay spectra of the metadevice at a selection of optical pump fluences from 0 to 1.4 mJ/cm^2^ without CW light illumination. (c) and (d) The modulated THz spectra at the shifted EIT resonance frequency with multiple optical pump fluences from 0 to 1.4 mJ/cm^2^ as the device is irradiated by a high CW light of 15.3 W/cm^2^.

### 2.2 CW light induced frequency-agility and optical pump excited modulations

We first demonstrate optically controlled EIT resonance frequency agility via the molecularization of THz metaatoms. To actively tune the resonance frequency, an external CW laser (808 nm) is utilized to induce the insulator-to-metal phase transition of the VO_2_-bridges with various light power densities. To compactly quantify the agile behavior of the resonance frequency, the normalized EIT resonances were evaluated as ${\delta T/\delta T}_{0\;\text{W}/{\text{cm}}^{2}}$ and ${\delta T/\delta T}_{15.3\;\text{W}/{\text{cm}}^{2}}$ for EIT-I and EIT-II, respectively. Here, δ*T* is the transmission difference between the EIT peak and dip. The normalized EIT resonances for both frequencies are traced as a function of the light power density. In Figure 2(a), the annihilation of EIT-I at 0.86 THz and the formation of EIT-II at 0.55 THz can be observed. With the increasement of power density, the frequency agility mainly occurs during the range of 4–8 W/m^2^. For a power density smaller than 8 W/m^2^, the EIT-I resonance strength remains almost unchanged due to the insulator state of the VO_2_-bridges. However, as the power density increases from 4 to 5.5 W/m^2^, the EIT-I resonance is annihilated rapidly from 100 to 0%, but no EIT resonance is observed at 0.55 THz. This result reveals that the conductivity enhancement at the beginning of the VO_2_ metallic transition can significantly suppress the dipole resonance in short CWRs and the LC mode in small SRRs, but fails to fully interconnect the gold unit structures. Additionally, in the range of 5.5–8 W/m^2^ an apparent occurrence of the EIT-II resonance resulting from the full molecularization of metaatoms is observed. For a more quantitative illustration, the THz dispersion spectrum evolution is also presented by applying a linearly increasing CW light power. For no light illumination, a pronounced EIT peak is discerned between two dips with a transmission of 89% at 0.86 THz, revealing that the EIT resonance amplitude δ*T*_EIT-I_ is as high as 44%. With the group delay amplitude defined as $\delta t_{\text{g}}=\Delta t_{\text{g}\;\text{EIT}\;\text{peak}}-\Delta t_{\text{g}\;\text{EIT}\;\text{dip}}$ , the corresponding δ*t*_g_ reaches 4.4 ps. As the power density of the CW light gradually increases to 5.47 W/m^2^, the EIT resonance undergoes a strong modulation to $\delta T_{\text{EIT}-\text{I}}=0\%$ and $\delta t_{\text{g}\;\text{EIT}-\text{I}}=4.4\;\text{ps}$ , as illustrated in Figure 2(b) and (c), demonstrating a complete elimination of EIT-I. At the next molecularization stage of the power density >5.5 W/m^2^, Figure 2(c) and (d) characterize the formation of EIT-II with a transparency window at 0.55 THz. The spectra of the transmission and group delay exhibit a strong EIT resonance feature with $\delta T_{\text{EIT}-\text{II}}=23\%$ and $\delta t_{\text{g}\;\text{EIT}-\text{II}}=2.4\;\text{ps}$ . Regarding the frequency-tuning range that is mathematically expressed as $\Delta f_{\text{I}\mathrm{–II}}/f_{\text{I}}$ [59, 60], the figure of merit of EIT frequency-tuning is quantitatively identified as 36%. The above observations provide significant evidence that the light-induced VO_2_ phase transition allows for an advanced operational frequency tuning as the resonant state changes between the atomized and molecularized metamolecules. As for the switching speed under the CW light stimulus, the rise time is approximately ∼2.2 s and the falling time is ∼4 s according to Ref. [61]. Please notice that the phase transition of VO_2_ leads to an extremely high conductivity change from ∼40 S/m to ∼2 × 10^5^ S/m in the THz regime, which is much larger than that of the amorphous Ge (from ∼10 S/m to ∼4 × 10^3^ S/m). Thus, a pure Ge film cannot realize the molecularization of EIT resonance.

**Figure 4:**
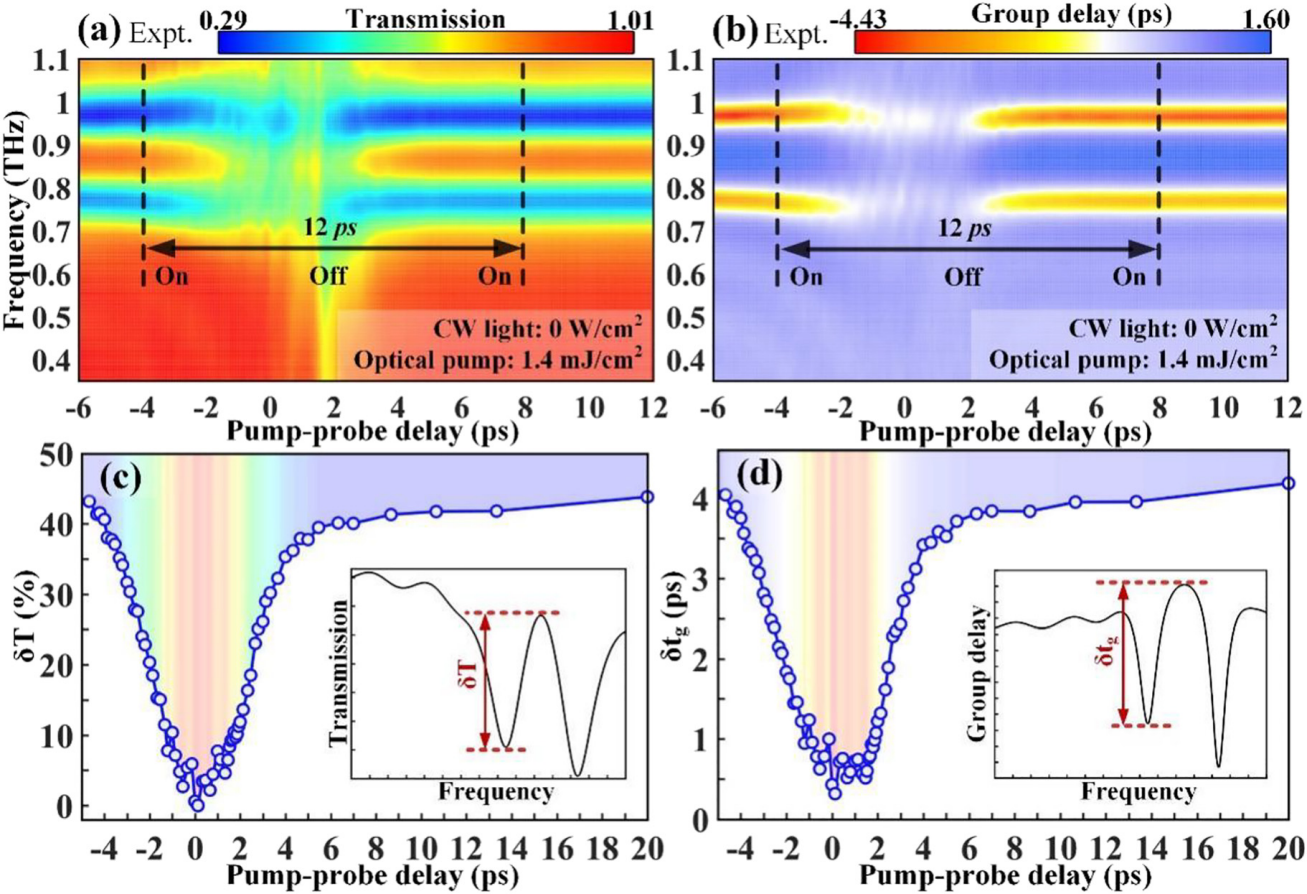
Experimental results of ultrafast and efficient photo-switching of THz wave at the original EIT resonance frequency of 0.86 THz. Monitored THz dispersion spectrum that is mapped as a function of the pump–probe time delay for (a) transmission amplitude and (b) group delay. (c) EIT resonance amplitude and (d) group delay amplitude versus the probe time delay showing transient modulation dynamics within one switching cycle. Here, the device is under no CW light illumination and the pump fluence remains a constant of 1.4 mJ/cm^2^.

**Figure 5:**
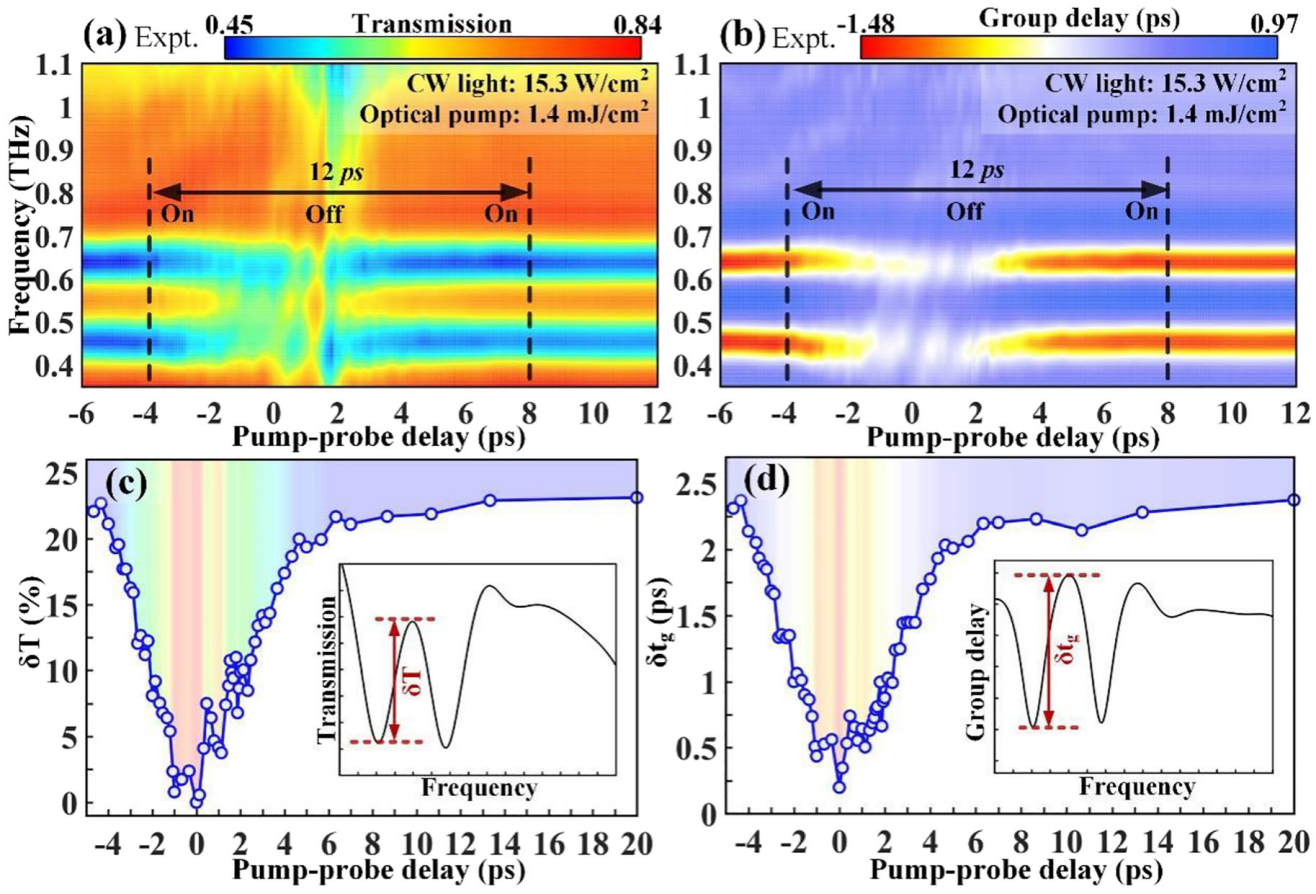
Experimental data of ultrafast and efficient photo-switching of THz wave at the shifted EIT resonance frequency of 0.55 THz after metaatom molecularization. The captured THz dispersion spectrum is mapped at different pump–probe time delays for (a) transmission amplitude and (b) group delay. (c) EIT resonance amplitude and (d) group delay amplitude versus the probe time delay showing transient switching dynamics within one switching cycle. Here, the device is under CW light illumination of 15.3 W/cm^2^ and the pump fluence remains constant at 1.4 mJ/cm^2^.

As the high light field localization in Fano-type metaatoms is susceptible to ambient environmental changes, the on-to-off active controlling of EIT resonance greatly fuels the era of ultrafast photoswitching electromagnetic waves, especially when a very limited photoconductivity can be induced [31, 42]. Here, we perform experimental measurements to inspect the ultrafast modulation behaviors for both fundamental and shifted EIT states by using femtosecond optical pulses. Owing to the time-dependent switching process, the THz probe pulses were scanned when the maximal modulation response occurred at the pump–probe time delay of 0 ps. Short-circuiting of the SRR gaps by photocarriers in amorphous Ge is the primary factor that causes the suppression of the dark mode, followed by the strong annihilation of the EIT windows. As depicted in Figure 3(a) and (b), the EIT-I resonance at 0.86 THz (no CW light is applied) gradually disappears with increasing optical pump fluence. After reaching saturation, the EIT-I resonance $\left(\delta T_{\text{EIT}-\text{I}}=44\%\text{and}\;\delta t_{\text{g}\;\text{EIT}-\text{I}}=4.4\;\text{ps}\right)$ is completely eliminated in the spectrum with $\delta T_{\text{EIT}-\text{I}}=0\%$ and $\delta t_{\text{g}\;\text{EIT}-\text{I}}=0.5\;\text{ps}$ at an average pump fluence of 1.4 mJ/cm^2^. Based on the working principle illustrated in Figure 1, the molecularization results in a reconfiguration from small to large SRRs, where capacitive gaps remain. The significance of this design can be recognized by noting the efficient switching-off of EIT-II resonance (CW light power density: 15.3 W/cm^2^) under optical pump injection, as shown in Figure 3(c) and (d). A pump fluence of 1.4 mJ/cm^2^ leads to a total suppression of EIT-II effect at a shifted frequency of 0.55 THz. According to the transmission modulation depth $D=\left(\delta T_{\text{no}\;\text{pump}}-\delta T_{\text{pump}}\right)/\delta T_{\text{no}\;\text{pump}}$ , its value can also be considered as 100% because $\delta T_{\text{EIT}-\text{II}}$ changes from 26 to 0%. Apart from the effective terahertz switching at selected frequencies, the results unequivocally confirm that there is no crosstalk between the frequency-agility response and the amplitude modulation response, implying the feasibility of dual-beam controlled multifunctionalities.

### 2.3 Ultrafast switching dynamics at CW light-controlled frequency-agile states

To show the picosecond-switching behavior of the frequency-agile EIT resonance at two states of atomized and molecularized metamolecules, the time-resolved terahertz spectra during the on–off switching period were monitored to capture the influence of the optical pumps. The ultrafast switching speed of the device is reliant on the lifetime of the photocarriers in the amorphous Ge layer evaporated on the sapphire substrate, and the photocarrier excitation and relaxation dynamics at a selection of pump fluences are shown in Figure S1. The femtosecond beam with a photon energy (1.55 eV) higher than the bandgap of the Ge film (0.66 eV) is utilized to pump the carriers from the valence band to the conduction band, resulting in dynamic photoconductivity. By unveiling the terahertz differential transmission (−Δ*E*/*E*_0_) as a function of the pump–probe time delay, we can identify the lifetime of charge carrier recombination using data fittings, which is described by a monoexponential decay equation convoluted with the instrument response function (IRF) [62]:

$$-\Delta E/E_{0}={\text{e}}^{-{\left(\frac{t-t_{0}}{\text{IRF}/2\;\text{ln}\;2}\right)}^{2}}\times \left(A_{0}+A_{1}{\text{e}}^{\frac{-t-t_{0}}{\tau_{1}}}\right)$$

where the IRF is determined by the pump pulse width to be ∼100 fs, the lifetime *τ*_1_ is the key parameter to be fitted, *A*_1_ and *A*_0_ denote the maximal fitted amplitude and the invariable basal signal, respectively, and *t*_0_ = 0 ps is the time constant for starting the decay fittings. As shown in Figure S1, the photoconductivity amplitude and relaxation lifetime by increasing the pump fluence are experimentally extracted, both showing conspicuous increases from 0.10 (598 fs) to 0.33 (813 fs). Unlike crystalline Si and Ge with a slow recovery time of the excited carriers (∼1 ms), a large number of defect states in the noncrystalline Ge film act as defect energy levels that greatly accelerate the carrier recombination rate, leading to an equilibration within a picosecond but with a very limited photoconductivity. The slower decay rate with a higher fluence is ascribed to the band-filling effect when trap-assisted recombination sites are overpopulated. To methodically delineate the ultrafast temporal evolutions of CW light-controlled operation frequency, we independently inspect the transient dynamics of EIT-I and EIT-II, which are adjusted by a CW light.

### 2.4 Picosecond timescale ultrafast switching of atomized metamolecules at 0.86 THz

Without exerting CW light, Figure 4 shows the transient evolution of atomized metamolecules when subjected to an optical pump with a fluence of 1.4 mJ/cm^2^. The false color maps in Figure 4(a) and (b) vividly confirm that the entire EIT switching on–off–on cycle is accomplished within ∼12 ps and the working frequency is centered at 0.86 THz. A detailed analysis of the resonance evolution was carried out by combining the captured terahertz spectra in Figure S2 at various pump–probe time delays. Specifically, the EIT-I resonance remains unaffected before −5.3 ps, which is significantly suppressed when the pump pulse starts to overlap the THz probe. At 0 ps with the maximal response, only the dipole mode is alive, while the transparency window is completely suppressed, representing the -off state. Next, the spectrum gradually restores to the original EIT-I resonance (dashed lines in Figure S2(c) and S2(d)), which almost recovers to the spectrum obtained at −5.3 ps after 13.3 ps of evolution. Therefore, the ultrafast and efficient EIT I resonance switching at the fundamental frequency is completed within 20 ps. Viewed from the perspective of time-resolved modulation depth, the EIT resonance and group delay amplitudes are extracted from the maps, as shown in Figure 4(c) and (d). Specifically, the EIT transmission amplitude at the -on state is approximately 44% before −5 ps, and then an apparent drop occurs, which reaches a dip of 0% at 0 ps, indicating a 100% modulation depth for EIT resonance. According to the definition of recovering rate $R=\delta T_{\text{pump}}/\delta T_{\text{no}\;\text{pump}}$ , *R* equals 95% at 10 ps. Since its value is very close to 100%, the resonance state can be approximately regarded as switching-on. The physically pertinent group delay undergoes a similar evolution process with its initial value of 4.2 ps decreased to 0.3 ps.

### 2.5 Picosecond timescale ultrafast switching of molecularized metamolecules at 0.55 THz

When the light power density is 15.3 W/cm^2^, the ultrafast switching dynamics are comparably studied in this section. The terahertz transmission/group delay against frequency and pump–probe time delay in the color maps have direct implications for the ultrafast on–off–on photoswitching cycle, as shown in Figure 5(a) and (b). The aspect starkly contrasted with the results without CW light is the working frequency. Thus, it is successfully to realize ultrafast all-optical switching of the EIT resonance at selective frequencies with one metadevice. The ultrafast switch is further visualized in Figure S3 where the dynamical spectrum evolution is observed. Similar to the ultrafast switching of EIT-I, the photoswitching at the shifted frequency is completed within 20 ps. The EIT resonance transmission and group delay amplitudes under ultrafast optical excitation are plotted in Figure 5(c) and (d), respectively, considering different pump–probe time delays. A 100% modulation depth for EIT-II resonance is also realized by suppressing the EIT transmission amplitude from 23 to 0% within 5 ps, and then recovering to 21% within 10 ps, indicating a recovering rate of 91%. Thus, we observe a systematic ultrafast photoswitching of EIT resonance at both fundamental and shifted frequencies within 20 ps and achieve unity modulation (*D* = 100%) with a pump fluence of 1.4 mJ/cm^2^.

### 2.6 Numerical simulations

To investigate the underlying physical origin of the aforementioned multifunctionalities, we performed a parametric study of the frequency-agile and amplitude modulation responses by sweeping the conductivity of the VO_2_-bridges or the Ge photoactive layer via numerical simulations. Notably, varying the conductivity of VO_2_ is equivalent to increasing the CW light power density, whereas the photoconductivity in Ge mimics the photocarriers generated by the femtosecond laser pump. According to the simulated far-field THz spectra in Figure S4, an apparent EIT resonance occurs at 0.88 THz when the VO_2_ bridges remain in an insulator state with 100 S/m. Owing to the phase transition, the metallic state with 2 × 10^5^ S/m achieves a broadband frequency shift of EIT (0.54 THz) with a tuning range of 39%. In addition to verifying the efficient terahertz switching via an optical pump, the elimination of the Fano peak at both fundamental and shifted frequencies as a function of Ge conductivity is also displayed in Figure S4. The significant agreement with the experimental data shown in Figure 3 further validates the effectiveness of optically active tuning of EIT metaatoms. Notably, metasurfaces supporting the EIT modes may cause a large enhancement of the local field, which plays a key role in the success of a high amplitude modulation depth.

**Figure 6:**
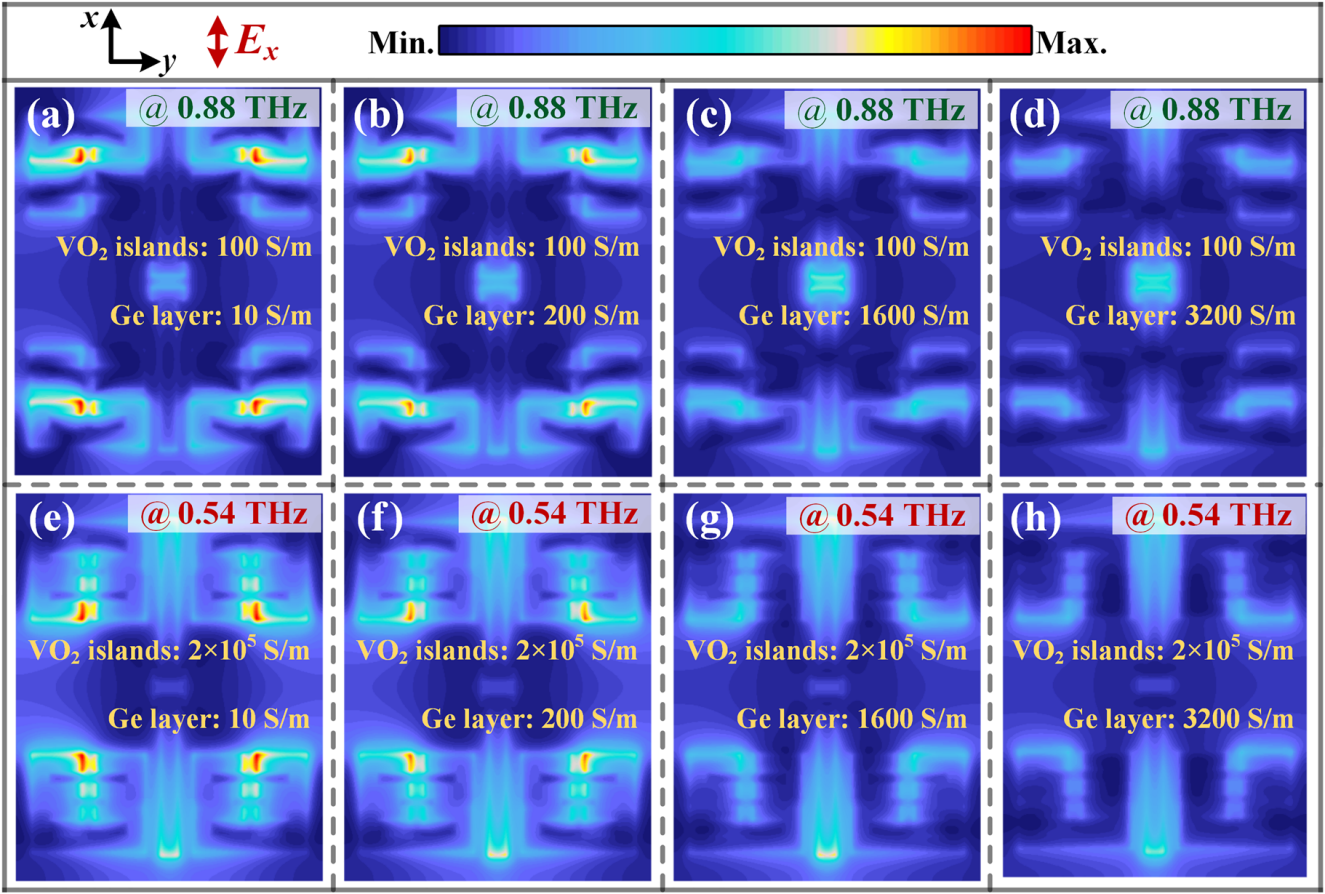
Near-field simulations for the proposed multifunctional metasurfaces by varying the conductivity of the Ge layer for (a)–(d) insulator and (e)–(f) metallic VO_2_ bridges, respectively.

These unique characteristics can be understood by an in-depth exploration of the near-field distributions at the EIT window peaks. The simulated field enhancement for different conductivities of Ge and VO_2_, is shown in Figure 6. Figure 6(a) and (e) show that the E-field enhancement positions change from small-SRRs’ gaps at 0.88 THz to large-SRRs’ gaps at 0.54 THz, indicating the completion of molecularization. At the EIT window point, the CWR serves as an electric dipole when excited by the ongoing electric field. The SRRs with a close eigenfrequency are then driven by the CWR via near-field coupling. The gaps serving as capacitors play a fundamental role in supporting the inductance–capacitance mode. Considering the extremely small gap size (<*λ*/68), capacitors with high field enhancement are inevitably easily short-circuited. With the increment of Ge conductivity, the E-field enhancement gradually drops, leaving only one electric dipole mode alive. Notably, the effective conductivity of the semi-metallic Ge film is much smaller than that of the metallic VO_2_-bridges, indicating the high sensitivity of our judiciously-engineered EIT resonances to weak photoconductivity. The parametric numerical study suggests that embedding different active materials into metaatoms would significantly improve the superior abilities of metaphotonic devices, which is helpful for practical applications of multifunctional plasmonic devices.

## 3 Results and discussion

In summary, we have experimentally demonstrated a newly multifunctional EIT platform. It is controlled by dual-optical approaches so that the ultrafast THz switching at frequency tunable channels is realized. The CW light can result in a phase transition of VO_2_-bridges, leading to a broadband EIT resonance frequency tuning behavior. Thus, changing the physical dimensions of metaatoms directly indicates an intriguing phenomenon in the application of multichannel data processing. By leveraging another degree of freedom, ultrafast modulation of resonant THz transmission is independently realized at different frequency channels. The tuning process is at picosecond timescale under femtosecond pulse excitation. This is attributed to the photocarriers with a sub-picosecond decay constant generated in the amorphous Ge film. Our proposed device is not limited to a single functionality in terms of a binary switchable frequency filter but is particularly suitable for ultrafast THz switching across a broad terahertz spectral range. Thus, it catalyzes the emergence of a novel all-optical metadevice by utilizing multiple beam properties. It also provides novel insights into the field of optoelectronic devices. Furthermore, the multiplexing characteristics of multifunctionalities may open up new avenues for the advancement of programmable metamaterials, spatiotemporal modulators, and multichannel high-speed terahertz switches, making them technologically disruptive in the development of active metasurfaces.

## 4 Experimental section

### 4.1 Device fabrication

A 100-nm-thick VO_2_ film with an intrinsic resistivity of 1 Ω cm at room temperature was grown on a 0001-oriented sapphire substrate in a radio-frequency plasma-assisted oxide molecular beam epitaxy (MBE) chamber. The fabrication of the hybrid metasurface was performed using a UV lithography system. We first attempted to define metallic metasurface structures patterned on the VO_2_ layer via the standard UV lithography technique. A 10-nm-thick Cr layer and a 200-nm-thick Au layer were sequentially deposited via electron beam evaporation, and then a lift-off process was carried out to fabricate the metaatoms. Then, the UV lithography and lift-off processes were repeated to define the VO_2_-bridges, and the redundant VO_2_ was removed through a reactive ion etching procedure. Subsequently, a 300-nm-thick germanium layer (thermal evaporation) was deposited on the entire surface to serve as the photoactive layer. According to the manufacturer, the intrinsic resistivity of germanium is 50 Ω cm. The area of the fabricated metaatoms was 6 × 6 mm^2^, which is much larger than the THz spot.

### 4.2 Terahertz transmission measurement

For the optical measurement of the sample, optical pump–terahertz probe equipment for terahertz spectroscopy measurements was selected. The femtosecond laser source (1 kHz repetition rate of 120 fs pulse width with a wavelength centered at 800 nm) was provided by a Ti:sapphire regenerative amplifier (Spectra-Physics). For sample excitation and THz pulse generation and detection, the femtosecond laser beam was split into three parts by beam splitters. One beam was passed through a translational stage that could change the optical path difference and was then inserted into the metadevice. The other was utilized to excite a 1-mm-thick (110)-oriented zinc telluride (ZnTe) crystal, and then terahertz pulses were generated based on the optical rectification effect. The terahertz spot has a diameter of ∼2.2 mm on the sample surface, where the optical pump beam (5 diameter) can uniformly cover the terahertz spot. Thus, the pump–probe time delay can be easily controlled by simply moving the translational stage. The third part of the femtosecond laser beam passing through another 1-mm-thick (110)-oriented ZnTe crystal was used to detect the terahertz time-domain waveforms according to the electro optic (EO) sampling technique. To obtain frequency-dependent terahertz dispersion spectra, a standard Fourier transformation was applied to the time-domain recorded THz electric field, and all spectra were normalized by dividing the THz spectrum obtained from a pure sapphire substrate (0001-oriented). In addition, to induce the phase transition of the VO_2_-bridges, an external CW light (808 nm) with a tunable power (0–5 W) was fed onto the surface of the sample by an optical fiber with a 45° angle. The diameter of the CW light on the metadevice surface was approximately 5 mm, which almost overlapped that of the optical pump beam.

### 4.3 Electromagnetic simulation

The numerical simulation was performed using finite element method (FEM) to determine the terahertz spectrum responses induced by the excitation of CW light and an optical pump. The Floquet boundary condition to indicate the periodically distributed metaatoms and perfectly matched layers (PML) to represent the infinite propagation distance are employed for the horizontal (*x*- and *y*-) and vertical (*z*-) directions in the 3D full electromagnetic simulations, respectively. The conductivities of the VO_2_-bridges and the Ge layer were optimized to match the experimental results obtained from experiments. The refractive index of the sapphire substrate used was considered as 3.4, which could be retracted from the TDS measurements according to the terahertz pulse time delay when the substrate was added. The metadevice was illuminated by an external plane wave with an *x*-polarized electric field 1 V/m above the device and the field propagated along the -*z*-direction. The transmission spectra were extracted below the metasurface and the near-field distributions were obtained within a one-unit cell area in the *xy*-plane just above the metasurfaces.

## Supplementary Material

Supplementary Material
