# Supplementary material for: Multidimensional engineered metasurface for ultrafast terahertz switching at frequency-agile channels
Source: Nanophotonics. 2022 Feb 22;11(7):1367–78. doi: 10.1515/nanoph-2021-0774 (PMC11501612; doi:10.1515/nanoph-2021-0774)
Supplement: Supplementary file 1 — Supplementary Material [file j_nanoph-2021-0774_suppl.docx]

**Supporting information**

Ultrafast Terahertz Switching at Frequency-Agile Channels in Multidimensional Engineered Metasurface

Yuze Hu,^1,2^ Mingyu Tong,^1^ Siyang Hu,^1^ Hao Hao,^3^ Weibao He,^1^ and Tian Jiang^1,2,*^

^1^ College of Advanced Interdisciplinary Studies, National University of Defense Technology, Changsha 410073, P. R. China

^2^ Beijing Institute for Advanced Study, National University of Defense Technology, Changsha 410073, P. R. China

^3^ College of Computer, National University of Defense Technology, Changsha 410073, P. R. China

E-mail: tjiang@nudt.edu.cn

The time dependence of the recovery of photoexcited free carriers in the pure 300-nm-thick amorphous Ge film deposited on a sapphire substrate is shown in Figure S1. The circled dots are the experimental data of time-resolved negative diﬀerence transmission obtained from OPTP measurements with several optical pump fluences. Solid lines are the results of fitting the corresponding dots with a single exponential decay dependence, indicating that the ultrafast relaxation is completed within several picoseconds.


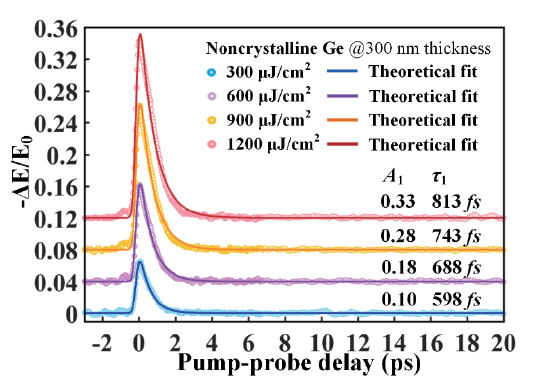


**Figure S1.** Photocarrier excitation and relaxation dynamics in the amorphous Ge layer evaporated on the sapphire substrate under diﬀerent pump fluences.


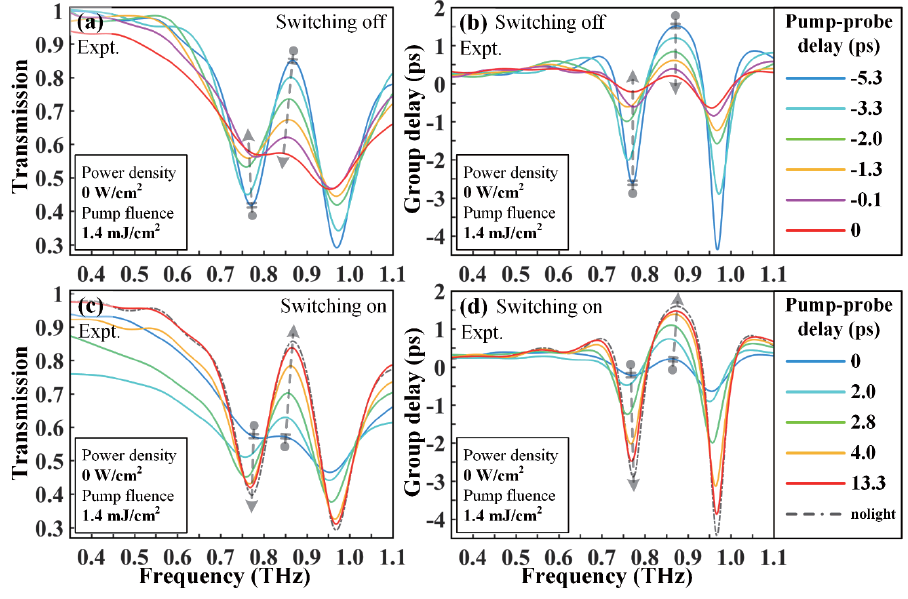


**Figure S2.** THz dispersion spectra captured from Figure 4 for the ultrafast switching of EIT resonance at the original frequency of 0.86 𝑇𝐻z. Experimental a) transmission and b) group delay spectrum evolution at various pump-probe time delays during the EIT switching-oﬀ process. c and d) The corresponding measured spectra during the EIT switching-on process recovering back to the unexcited EIT state. The dotted arrow indicates the evolution trend during the switching processes.


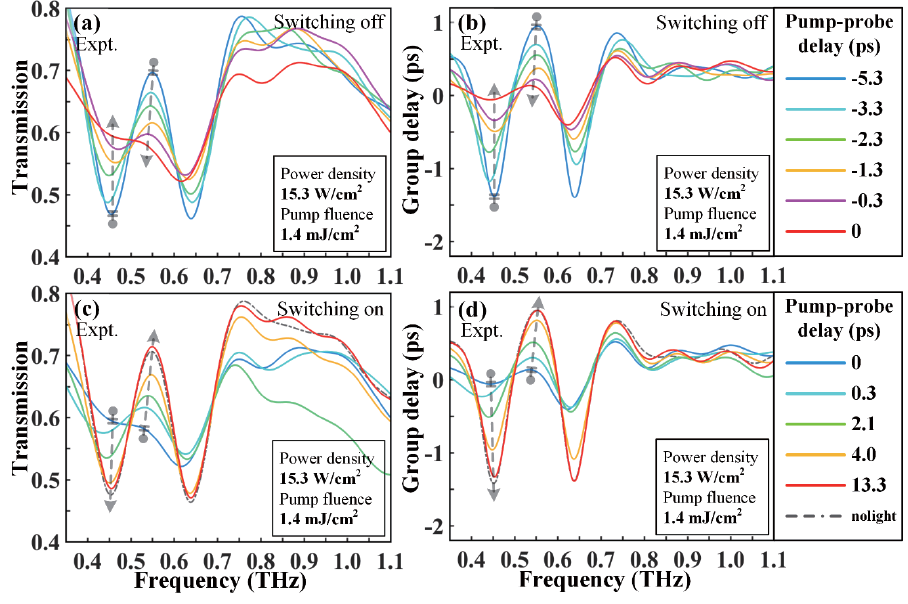


**Figure S3.** THz dispersion spectra extracted from Figure 5 for the ultrafast switching of EIT resonance at the shifted frequency of 0.55 𝑇𝐻z. Experimental a) transmission and b) group delay spectra are plotted as a function of pump-probe time delay showing the EIT switching-oﬀ process. c) and d) The corresponding experimental spectra during the EIT switching-on process demonstrating recovery to the unexcited EIT state. The dotted arrow indicates the evolution trend during the switching processes.


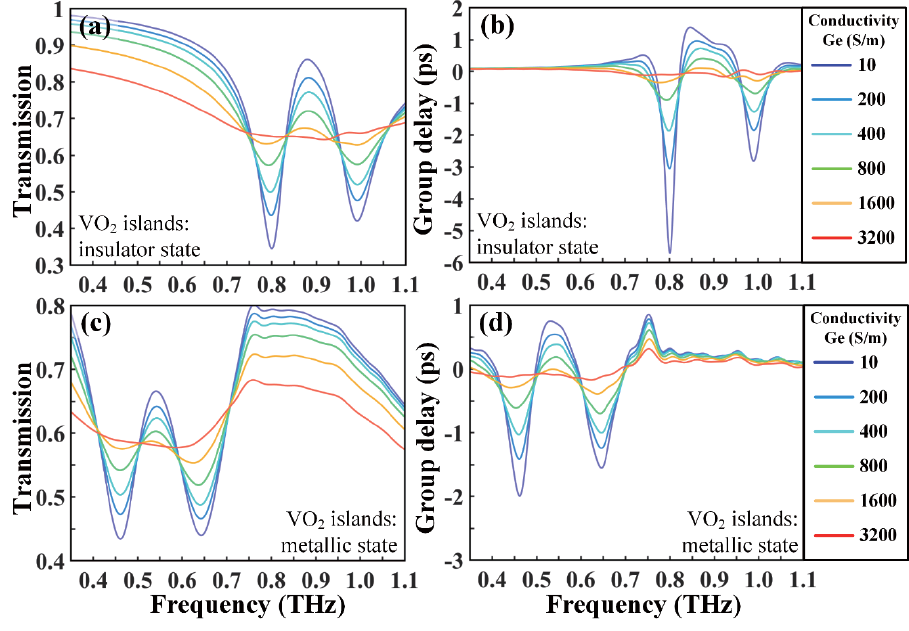


**Figure S4.** Spectral analysis of EIT responses under optical pump injection by changing the photoconductivity in the Ge layer before and after metaatom molecularization, respectively. Simulated THz transmission and group delay spectra with varying conductivity of the Ge layer from 1 to 3200 𝑆/m when a) and b) the VO_2_-bridges stay in the insulator state, indicating that no molecularization occurs; and c) and d) with the VO_2_-bridges phase changed to the metallic state, implying that the metaatoms were molecularized.
